# Effect of PF-02341066 and radiation on non-small cell lung cancer cells

**DOI:** 10.3892/or.2012.2198

**Published:** 2012-12-18

**Authors:** VASU TUMATI, SUBASHRI KUMAR, LAN YU, BENJAMIN CHEN, HAK CHOY, DEBABRATA SAHA

**Affiliations:** 1 Department of Radiation Oncology, University of Texas Southwestern Medical Center, Dallas, TX 75390, USA; 2 Simmons Comprehensive Cancer Center, Dallas, TX 75390, USA

**Keywords:** radiation resistance, radio-sensitization, non-small cell lung cancer, ALK inhibitors, DNA double-strand break repair, animal models, tumor growth delay

## Abstract

Recently, a fusion protein of echinoderm microtubule associated protein like-4 (EML4) and anaplastic lymphoma kinase (ALK) has been found in non-small cell lung cancer (NSCLC) patients. In addition, endogenous expression of phosphorylated c-Met was found to be increased in many invasive NSCLC cases. PF-02341066 (crizotinib) is a novel dual c-Met and EML4-ALK inhibitor, and preclinical studies have shown that treatment with ALK inhibitors leads to drastic tumor regression in xenograft models. A phase I trial of PF-02341066 yielded a 53% response rate and a disease control rate of 79%. We evaluated crizotinib as a potential radiation-sensitizing agent in multiple established NSCLC cell lines with varying expression levels of c-Met and EML4-ALK. The combined effect of ionizing radiation (IR) and PF-02341066 was determined by the surviving cell fraction, cell cycle distribution, apoptosis, DNA double-strand break repair in 5 NSCLC cell lines (A549, H460, H3122, H2228 and H1993) and in *in vivo* xenograft studies. Treatment of NSCLC cells with either PF-02341066 alone or PF-02341066 + IR did not significantly alter cellular radiosensitivity, DNA repair kinetics and cell cycle distribution; no significant enhancement of tumor growth delay was noted in response to the combined treatment of PF-02341066 + IR. EML4-ALK and c-Met inhibition leads to activation of parallel pathways that converge on Akt signaling which abrogates any radiation-sensitizing effect. Although PF-02341066 is an effective therapy able to suppress tumor growth in tumors that exhibit positivity for either EML4-ALK or c-Met, it did not affect the intrinsic radiation response of tumor cell lines. In the present study, we demonstrated that PF-02341066 did not enhance radiation sensitivity in a panel of NSCLC cell lines.

## Introduction

Lung cancer is a leading cause of cancer-related death both in the US and worldwide. NSCLC represents 80% of lung cancers. It is estimated that 40% of patients who present with NSCLC are already at an advanced inoperable stage of the disease (1). For those patients who present with late stage disease, therapy consists of a combination of radiation and chemotherapy. Unfortunately, many NSCLC subpopulations exhibit intrinsic radiation resistance which leads to local recurrence, lymphovascular invasion and distant metastatic disease.

A fusion protein of echinoderm microtubule associated protein like-4 (EML4) and anaplastic lymphoma kinase (ALK) has been found in NSCLC patients (2). It is estimated that approximately 5% of NSCLC cases harbor an EML4-ALK fusion (3,4). The EML4-ALK fusion functions in a manner similar to EGFR mutations; that is, EML4-ALK constitutively activates a tyrosine kinase receptor leading to cancer dependence on overactive mitogenic pathways (5). Transgenic mice that express the EML4-ALK fusion protein grow numerous lung adenocarcinomas (6). This constitutive mechanism represents a prime target for chemotherapy.

PF-02341066, a novel dual c-Met and ALK inhibitor, has recently been evaluated in both preclinical and clinical trials. Preclinical studies have shown that treatment with ALK inhibitors can lead to drastic tumor regression in *in vivo* xenograft models (7). A phase I trial of PF-02341066 revealed impressive results with a 53% response rate and a disease control rate of 79% (3). PF-02341066 is currently under evaluation as a secondary agent as well as a single-drug therapy in phase III and phase II trials, respectively.

While PF-02341066 has shown significant and promising results as a chemotherapeutic agent, it has not been evaluated, to date, in conjunction with radiation in NSCLC models. In this study, we evaluated PF-02341066 as a potential radiation-sensitizing agent in 5 different established NSCLC cell lines (H460, A549, H3122, H2228 and H1993) with varying expression levels of c-Met and EML4-ALK (8).

## Materials and methods

### Cell culture and reagents

Human NSCLC cell lines H460, A549, H3122, H1993 and H2228 were kindly provided by Dr John D. Minna at the UT Southwestern Medical Center, Dallas, TX. These cell lines were maintained in RPMI-1640 with 10% FBS and 50 units/ml penicillin and 50 μg/ml streptomycin in 5% carbon dioxide at 37°C.

PF-02341066 (MW, 450.3) was obtained from Pfizer Inc., dissolved in DMSO to give a stock solution of 10 mM and stored at −20°C. Cells were irradiated using a ^137^Cs source (Mark 1–68 irradiator, J.L. Shepherd and Associates, San Fernando, CA) at a dose rate of 3.47 Gy/min (9).

### Clonogenic survival assay

Exponentially growing cells were treated with PF-02341066 for 2 h and then treated with increasing doses of IR (0, 2, 4, 6 and 8 Gy). Cells were trypsinized and counted using a particle counter (Beckman Coulter, Inc.), diluted serially to appropriate concentrations and plated into a 60-mm dish in triplicate. After 7 or 14 days of incubation, the colonies were fixed and stained with 4% formaldehyde in PBS containing 0.05% crystal violet. Colonies containing >50 cells were counted. The surviving cell fraction was calculated as: (Mean colony counts)/[(cells inoculated) × (plating efficiency)], in which plating efficiency was defined as (Mean colony counts)/(cells inoculated for unirradiated controls). The data are presented as the mean ± SD of at least 3 independent experiments. The curve S = e ^−(αD + βD2)^ was fitted to the experimental data using a least square fit algorithm using the program SigmaPlot (Systat Software, Inc.) as previously described (9). The radiation dose enhancement ratio (DER) was calculated as the dose (Gy) for radiation alone divided by the dose (Gy) for radiation plus drugs (normalized for drug toxicity) resulting in a surviving cell fraction of 0.25. Clonogenic survival assay was also performed to determine the growth inhibitory response (50%) of these NSCLC cells using increasing dosages of PF-02341066. Inhibitory dose concentrations were determined using a 4 parameter variable slope regression model.

### Immunoblot assay

Cell lysates were prepared from each sample as previously described (10). An equal amount of total protein (20 μg) was subjected to a 10% SDS-PAGE for immunoblot analysis and probed with primary antibodies as indicated. β-actin was used for the loading control.

### Cell cycle analysis

Cell cycle assays were performed with propidium iodide (PI, 100 μg/ml) as previously described (10). At least 20,000 cells were counted; the proportion of cells of different phases was gated and calculated using the software FlowJo 8.7.1 (Tree Star, Inc.).

### DNA double-strand break (DSB) repair assay

DSB repair assay was performed by counting phospho-γH2AX foci following treatment with IR alone, drug alone or IR + PF-02341066, as previously described (10). Cells were plated on poly-lysine-coated coverslips and were allowed to attach and then treated as indicated. Cells were fixed in 4% formaldehyde/PBS for 30 min, permeabilized in 0.5% Triton X-100 in PBS for 1 h, and blocked in 5% bovine serum albumin and 1% normal goat serum for 1 h at room temperature. Cells were then incubated with the primary antibody, anti-phospho-Histone γH2AX (Ser139; 1:2,000) for 1 h. Rhodamine red-conjugated goat anti-mouse was used as a secondary antibody. Cells were mounted in a Vectashield mounting medium containing 4′,6-diamidino-2-phenylindole (DAPI). Phospho-γH2AX foci were examined using a fluorescence microscope (CRG Precision Electronics). The number of phospho-γH2AX foci was determined at each time point (average of 50 nuclei), and the percentage of remaining foci was plotted against time to obtain DSB repair kinetics. Data are represented as the mean ± SEM.

### Tumor growth delay (TGD)

Female athymic nude mice (nu/nu, 5–6 weeks) were injected (1×10^6^ cells in 100 μl) s.c. into the right posterior flanks. Treatment was initiated when tumors reached a diameter 2–3 mm in size. Treatment groups (5 animals each) included untreated control (0.9% saline), PF-02341066 alone (50 mg/kg/day for 5 days, p.o.), radiation [2 Gy/day, 5 days, X-RAD 320 (Precision X-Ray, North Branford, CT] and combined treatment of PF-02341066 and irradiation. The drug was administered 1 h before radiation. Tumors were measured 3 times per week using a Vernier caliper. Results were evaluated with the formula: Volume = 0.5abc (a, width, b, length and c, thickness). Tumor growth delay (TGD) was calculated as the time for treated tumors to reach 1,000 mm^3^ minus the time for control tumors to reach the same volume. Enhancement factor (EF) was then determined as follows: EF = (TGD_drug + IR_ - GD_drug_)/GD_IR_ as previously described (10,11). All the experiments were conducted under the Institutional Animal Care and Use Committee of UT Southwestern Medical Center, Dallas, TX approved guidelines for animal welfare.

### Apoptosis assay

Cells were plated in a 100-mm dish and 24 h later were treated with IR alone, drug alone or IR + drug. Floating and attached cells were harvested post treatment as indicated. After centrifugation (200 × g, 5 min), the medium was removed, and the cell pellet was carefully resuspended in 5 ml PBS. Phycoerythrin (PE) Annexin V apoptosis detection kit I (BD Biosciences) was used to identify apoptotic cells by flow cytometry. Cells that stained positive for PE Annexin V and negative for 7-AAD (right bottom quadrant) were undergoing apoptosis. The proportion of apoptotic cells were gated and calculated by FlowJo 8.7.1 as previously described (10).

### Statistical analysis

Data are presented as the means ± SD or SEM, as noted, of at least 3 independent experiments. Results were tested for significance using either Mantel-Cox log-rank test, Mann-Whitney rank sum test, or t-test as noted.

## Results

### Specificity of PF-02341066 in NSCLC

**Figure 1:**
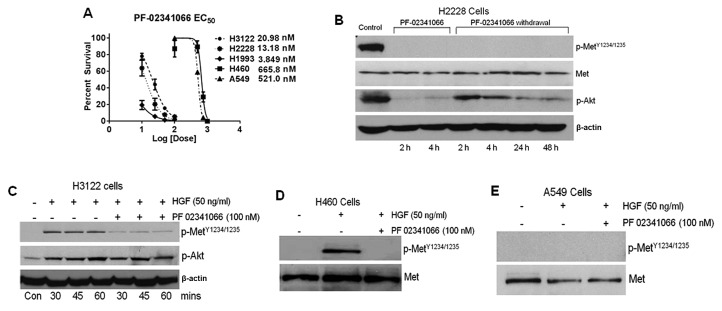
Effect of PF-02341066 on NSCLC cell lines. (A) Concentration of PF-02341066 necessary to inhibit clonogenic growth by 50% was determined by clonogenic surviving assay. (B) H2228 cells were treated with PF-02341066 for 4 h after which the drug was removed from the culturing medium. Samples were collected at the times noted. (C-E) H3122, H460 and A549 cells were treated with HGF (50 ng/ml) in the presence or absence of PF-02341066, and samples were collected as indicated for western blot analysis.

The toxicity of PF-02341066 was reported at 50% colony survival and determined in a set of several NSCLC cell lines. Drug toxicity varied greatly among the cell lines; H2228 (13.18 nM), H3122 (20.98 nM) and H1993 (3.85 nM) were highly sensitive to PF-02341066 whereas, H460 (666 nM) and A549 (521 nM) were resistant (Fig. 1A).

PF-02341066 specificity was then determined by the phosphorylation status of the c-Met receptor at Tyr^1234/1235^ residues before and after treatment with HGF using immunoblot analysis. For this study, H3122 and H2228 cells were used specifically because of their differential level of endogenous phosphorylated c-Met as shown in Fig. 1B and C. H3122 cells demonstrated induction of phosphorylation within 30 min after addition of HGF (Fig. 1C). HGF-induced c-Met phosphorylation in H3122 was completely inhibited when the drug was added 2 h prior to the addition of HGF. Akt phosphorylation was also increased upon addition of HGF, however, treatment with PF-02341066 did not block Akt phosphorylation. The endogenous level of phospho-c-Met was significantly higher in H2228 cells and there was no further enhancement of phosphorylation after addition of HGF (Fig. 1B). PF-02341066 completely prevented endogenous c-Met phosphorylation in H2228 cells within 2 h. In addition, the c-Met receptor remained unphosphorylated up to 48 h after removal of the drug from the medium. However, Akt, which is a downstream target of c-Met, displayed a high level of phosphorylation in H2228 cells; p-Akt levels were reduced by PF-02341066 treatment. phospho-Akt, did, however, reappear after removal of the drug. Furthermore, a small amount of pAkt was noted after a 4-h drug treatment. PF-02341066 also prevented c-Met phosphorylation in H460 cells (Fig. 1D). HGF-mediated induction of c-Met phosphorylation was not observed in A549 cells (Fig. 1E). These results are also summarized in Table IA.

### PF-02341066 does not affect radiation sensitivity in vitro or in vivo

**Figure 2:**
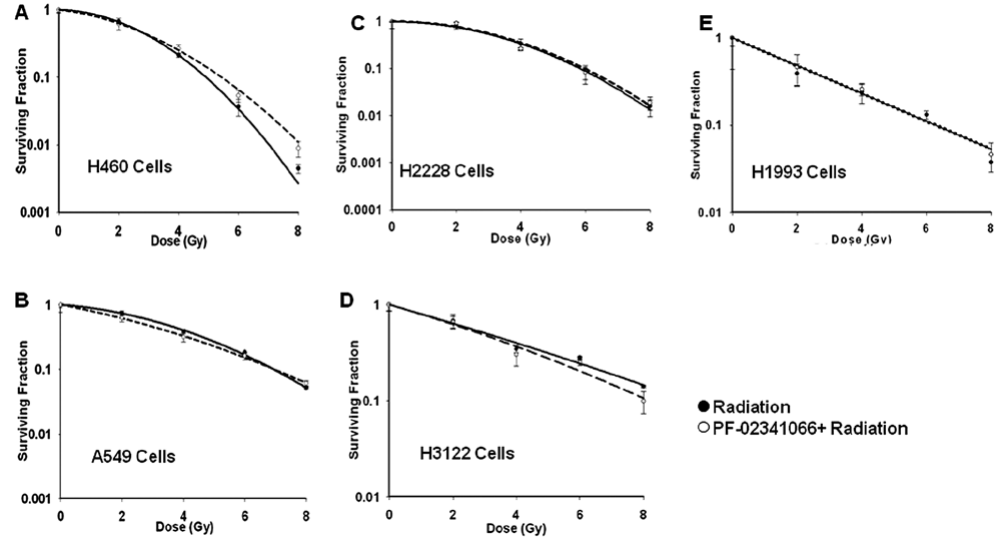
Effect of radiation and PF-02341066 on NSCLC cells. Cells were grown to 70% confluence prior to treatment with PF-02341066 for 2 h and then cells were irradiated with increasing doses of radiation (2, 4, 6 and 8 Gy). Cells were then washed, trypsinized and plated for colony formation assay. (A) H460 cells (100 nM); (B) A549 cells (100 nM); (C) H2228 cells (13 nM); (D) H3122 cells (21 nM) and (E) H1993 cells (3.8 nM).

The modulation of radiation sensitivity by PF-02341066 was investigated in all 5 NSCLC cell lines (Fig. 2). Cells were treated with the drug for 2 h before being treated with increasing doses of IR (0, 2, 4, 6 and 8 Gy). The maximally tolerated dose used in phase II trials of PF-02341066 resulted in trough plasma concentrations of 57 nM (12); therefore, cell lines with EC_50_ below the MTD were treated at the EC_50_ concentration while cell lines that were resistant were treated at 100 nM; slightly less than twice the clinically relevant concentration. Intrinsic radiation response of each cell line was different (Fig. 2) and their corresponding SF_2_ values are shown in Table I. Notably, when these cells were treated with PF-02341066 followed by IR, very little or no change was noted between treated and untreated SF_2_ values (Table I) for any of these cell lines (P>0.05 for all SF_2_ data). Further assays were performed in which cells were either treated concurrently or sequentially with radiation prior to PF-02341066 to modulate the sensitivity; however, no enhancement was observed regardless of the treatment condition (data not shown).

**Figure 3:**
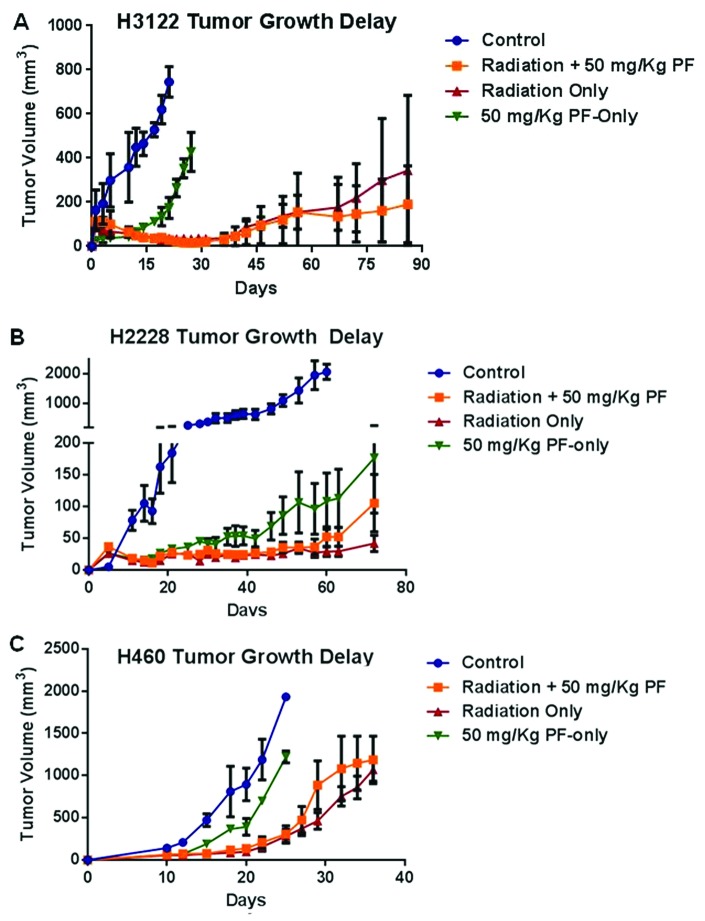
Effect of radiation and PF-02341066 on NSCLC xenografts. (A and B) One million cells in 100 μl volume with Matrigel (1:1) were injected subcutaneously into female nude mice. Mice were divided into the following treatment groups: control, PF-02341066 only (50 mg/kg), IR (2 Gy × 5) and IR + PF 02341066. Treatments were initiated when the tumor diameter was 3–5 mm. (C) H460 cells, which are non-responsive to PF-02341066 treatment, were injected into female nude mice without Matrigel and the tumor-bearing animals were divided into 4 groups and treated as described above.

Next, the radiation sensitivity modulation by PF-02341066 was studied in *in vivo* xenograft models of 3 different cell lines, H3122, H2228 and H460 (Fig. 3). H3122 tumors that were treated with IR (2 Gy × 5) or IR (2 Gy × 5) plus PF-02341066 (50 mg/kg, recommended dose) showed significant growth delay; however, no difference was appreciated between the two groups (Fig. 3A). It was also noted that following treatment with PF-02341066 alone, the time for a tumor to reach 500 mm^3^ was significantly different when compared with the control (28 vs. 15 days, respectively) (P=0.01). The H2228 *in vivo* experiment, following treatment with IR and IR + PF-02341066 yielded similar results as H3122 with significant growth arrest noted with no appreciable difference between the IR and IR + drug groups (Fig. 3B). However, treatment with PF-02341066 alone showed significant TGD in the H2228 xenografts (Fig. 3B). These *in vivo* results further recapitulate the *in vitro* surviving fraction data which showed that no significant radiation dose enhancement occurred when PF-02341066 was added to the treatment. The drug resistant cell line, H460, was also tested in the xenograft experiment. Treatment with IR alone and IR plus PF-02341066 resulted in TGD of 30 and 27 days, respectively (Fig. 3C). The effect of radiation and radiation plus drug was not significantly different (P=0.58). It is important to note that in the H460 model, the effect of radiation treatment was significantly different than treatment with PF-02341066 alone (P=0.008).

### Effect of PF-02341066 on IR-induced DNA repair kinetics

**Figure 4:**
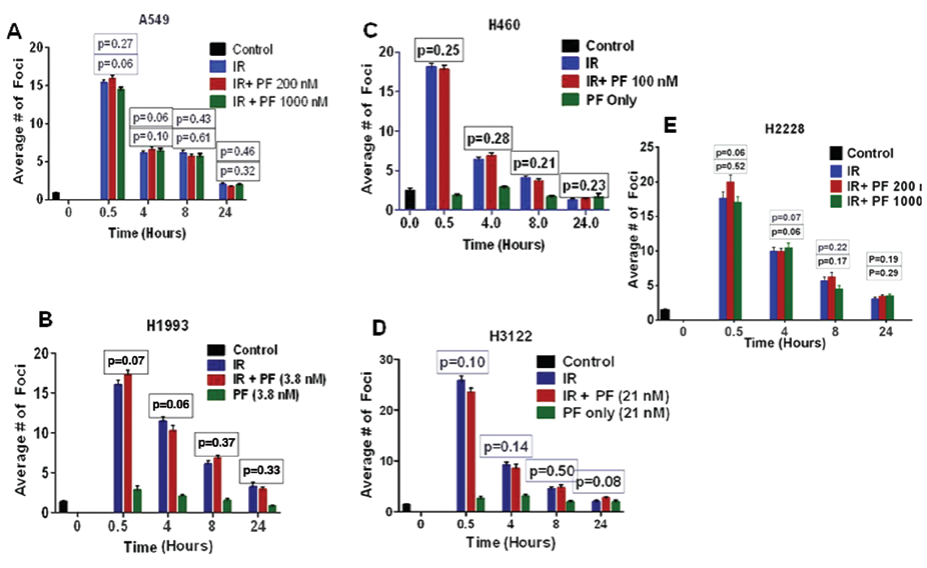
Effect of PF-02341066 on radiation-induced DNA-double-strand break repair. Cells were treated with varying concentrations of PF-02341066 as indicated, irradiated with 2 Gy, and fixed in paraformaldehyde at the designated time points for H2AX staining. (A) A549; (B) H1933; (C) H460; (D) H3122 and (E) H2228 cells.

Previous reports have suggested that c-Met and Alk kinases provide a survival advantage to cancer cells through their effects on DNA DSB repair (13–15). Therefore, c-Met inhibition leads to changes in DNA double-strand break (DSB) repair kinetics (Fig. 4). To measure the effect of PF-02341066 on the repair of IR-induced DNA DSB repair, the different NSCLC cell lines were exposed to 2 Gy of radiation and fixed in paraformaldehyde at the times indicated for γH2AX staining (Fig. 4). c-Met/EML4-ALK inhibition by this drug did not induce foci formation in any of the cell lines tested (Fig. 4). Previous reports with different c-Met inhibitors have shown radiation-sensitizing effects in glioma models. To examine whether the previously reported effect could be replicated with supra-physiologic doses, we treated A549, a non-responding cell line, and H2228, a c-Met/EML4-Alk-positive cell line, with various high doses of PF-02341066 (Fig. 4A and E). Treatment with this drug, regardless of dose, did not modify IR-induced DNA DSB repair kinetics in any of the cell lines tested in this study (Fig. 4).

### Effect of PF-02341066 and radiation on cell cycle arrest in NSCLC cells

**Figure 5:**
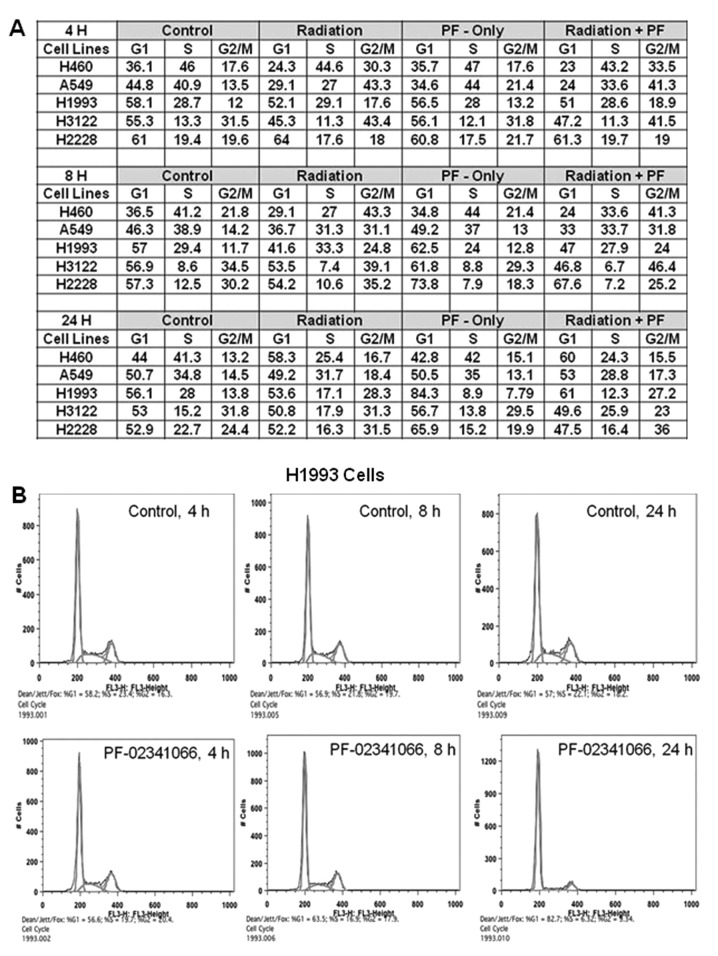
Effect of PF-02341066 and radiation on the cell cycle distribution. Cells were plated and treated as previously described. The radiation groups received 2 Gy radiation while the drug treatment groups were treated at the cell line-specific EC_50_ values. (A) All cell lines show typical G2/M arrest associated with ionizing radiation, and addition of PF-02341066 failed to increase the proportion of cells that were arrested. H1993 and H2228 cells showed strong G1 arrest following treatment of PF-02341066 only. (B) Representative flow cytometric data from H1993 cells. Cells that were treated with 3.8 nM PF progressively underwent G1 arrest with the greatest effect noted at 24 h.

In this experiment each cell line showed increased G2/M arrest in response to IR at 4 and 8 h (Fig. 5). The effect of the combined treatment of PF-02341066 and IR did not significantly differ from that of IR alone in any of the cell lines with respect to G2/M arrest. It is interesting to note that in cell lines that expressed endogenous phospho-c-Met, H2228 and H1993, drug treatment alone induced G1 arrest (Fig. 5). The G1 arrest was most pronounced at 8 h for H2228 cells (57–74%) and 24 h for H1993 cells (56–84%). In H2228 cells, it appeared that G1 arrest attenuated the G2M arrest normally observed with radiation alone. This result was further verified in H2228 cells by demonstrating a decreased number of cells in M-phase after treatment with PF-02341066 using phospho-Histone3 analysis (data not shown).

### Combination therapy results in additive increases in apoptosis

It was previously shown that cells containing ALK fusions undergo increased apoptosis following a 48-h PF-02341066 treatment (16). Therefore, we aimed to ascertain whether the combination of radiation and PF-02341066 has an additive or supra-additive effect on apoptosis. In those cells considered resistant to PF-02341066, H460 and A549, 48 h of treatment produced little apoptosis: 1.3 and 5.9%, respectively, when compared to the untreated samples, while an additive increase in apoptosis was noted when PF-02341066 was combined with radiation. In A549 cells, this additivity was achieved at concentrations of drug greater than clinically achievable. In H1993, H3122 and H2228 cells, a greater degree of apoptosis was noted; 2.25, 5.31 and 5.9%, respectively. However, when PF-02341066 was combined with radiation the percentage of apoptosis noted was roughly additive (Table IB).

## Discussion

c-Met activity has been associated with increased radiation resistance (17,18). Furthermore c-Met is known to be an upstream activator of Akt which has also been linked to radiation resistance (15,19). In addition, recent studies have implied that the EML4-ALK fusion protein may interact with many similar pathways similar to c-Met (13,14). We, therefore, investigated whether c-Met and EML4-ALK inhibition by PF-02341066 leads to increased radiation sensitivity in NSCLC cells.

Initially, we determined the toxicity of PF-02341066 in a panel of NSCLC cell lines (Fig. 1A). It became apparent that the cell lines could be divided into responders (H2228, H1993 and H3122) and non-responders (A549 and H460). Not surprisingly, the c-Met-positive cell lines H2228 (which is also EML4-ALK-positive) and H1993 responded well to PF-02341066 treatment. Among the non-responding cell lines, H460 has been classified as EML4-ALK-positive but contains a different variant than that of the H2228 and H3122 cell lines (8). However, the classification of H460 as an EML4-ALK carrier remains controversial with some studies failing to find this fusion (20). It is important to note that the EC_50_ concentrations of the non-responding cell lines were well above the maximally tolerated dose (plasma concentration of 57 nM) as determined in phase II trials of PF-02341066 (12). In addition, although we used a concentration of 100 nM for treatment, we confirmed that this dose did effectively inhibit c-Met phosphorylation (Fig. 1D).

We clearly demonstrated that, within the context of the cell lines we studied, treatment with PF-02341066 did not increase radiation sensitivity. For 5 cell lines used in this study H2228, H3122, H1993, H460 and A549; there was no significant dose enhancement *in vitro*. When tested in *in vivo* models, there was statistically significant increase in radiation response with treatment. The dose used in this experiment, 50 mg/kg, has been shown to be the cut-off dose in which further dose escalation results in marginal increases in plasma concentration (7). c-Met has been implicated in modulating DNA repair kinetics which is thought to provide a survival advantage to cancer cells (15). However, we showed that c-Met inhibition through PF-02341066 does not affect DNA repair kinetics. These data are further supported by cell cycle analysis which failed to show an increased proportion of cells in G2M arrest indicating that cells that are damaged are able to repair at the same rate regardless of c-Met inhibition. It is interesting to note that H2228 and H1993 cells showed increased G1 arrest following treatment of the drug alone; however, this increased G1 arrest did not result in significant changes in radiation response. The G1 arrest noted for H2228 and H1993 cells represents the most likely mechanism for decreased clonogenicity.

Finally, we assayed for PF-02341066 specificity *in vivo*. We report that in H2228, a cell line that has high levels of endogenous c-Met, PF-02341066 irreversibly inhibits c-Met activity. We showed, however, that Akt phosphorylation was only transiently affected and phosphorylation reappeared after 4 h of treatment. In addition, once the drug was removed from the medium there was a strong p-Akt rebound. In the cell lines that did not express endogenous phospho-c-Met, HGF did induce c-Met phosphorylation which was inhibited by PF-02341066. However, in these cell lines, the level of p-Akt was largely unaffected by treatment. The inability to keep p-Akt suppressed throughout the treatment course may represent an escape pathway for these NSCLC cells. Although we were inhibiting an upstream modulator of Akt, it appeared that the cells were able to compensate through separate and redundant pathways thereby reactivating the Akt pathway, a pathway known to increase radiation resistance.

Even though PF-02341066 is an effective therapy able to suppress tumor growth in NSCLC tumors that are either EML4-ALK- or c-Met-positive, it did not affect tumor cell radiation resistance in any appreciable manner. We showed that PF-02341066 did not affect radiation sensitivity, DNA repair kinetics, the cell cycle distribution or increased apoptosis in a supra-additive manner. PF-02341066 may be able to escape the radiation-sensitizing effects of c-Met inhibition by reactivating the Akt pathway further downstream or through a separate and redundant pathway.

## Figures and Tables

**Table I:** Characterization and cellular apoptosis of NSCLC cells following IR and PF-02341066 treatment.

A, Characterization of NSCLC cells						

Cells	EML4-Alk fusion	LD_50_ (nM)	SF_2_	DER	Endogenous pc-Met^a^	HGF inducible pc-Met^a^
H1993	Negative	3.90	0.48	1.00	+	No
H2228	Positive	13.18	0.76	1.00	++	No
H3122	Positive	20.98	0.64	1.10	-	Yes
H460	Positive	665.80	0.54	0.95	-	Yes
A549	Negative	521.00	0.73	1.08	-	No

B, Cellular apoptosis of NSCLC cells						

Treatment		H460	A549	H3122	H1993	H2228

Control		0.89	1.84	1.19	2.09	2.44
Radiation		9.83	4.01	3.43	6.28	13.4
PF-02341066 alone		1.54	5.09	1.72	4.65	5.37
Radiation + PF-02341066		7.32	12.07	4.93	6.69	19.74

a pc-Met, Y^1234/1235^; SF_2_, surviving fraction at 2 Gy; DER, dose enhancement ratio at 0.25 survival.
